# Disruption of miRNA sequences by TALENs and CRISPR/Cas9 induces varied lengths of miRNA production

**DOI:** 10.1111/pbi.13315

**Published:** 2019-12-20

**Authors:** Honghao Bi, Qili Fei, Riqing Li, Bo Liu, Rui Xia, Si Nian Char, Blake C. Meyers, Bing Yang

**Affiliations:** ^1^ Department of Genetics, Development and Cell Biology Iowa State University Ames IA USA; ^2^ Department of Plant and Soil Sciences Delaware Biotechnology Institute University of Delaware Newark DE USA; ^3^ Donald Danforth Plant Science Center St. Louis MO USA; ^4^ Division of Plant Sciences University of Missouri Columbia MO USA; ^5^ State Key Laboratory for Conservation and Utilization of Subtropical Agro‐Bioresources College of Horticulture South China Agricultural University Guangzhou Guangdong China; ^6^ Present address: Agricultural Genomics Institute at Shenzhen The Chinese Academy of Agricultural Sciences Shenzhen China

**Keywords:** rice, Arabidopsis, TALENs, CRISPR/Cas9, gene editing, miRNA

## Abstract

MicroRNAs (miRNAs) are 20‐24 nucleotides (nt) small RNAs functioning in eukaryotes. The length and sequence of miRNAs are not only related to the biogenesis of miRNAs but are also important for downstream physiological processes like ta‐siRNA production. To investigate these roles, it is informative to create small mutations within mature miRNA sequences. We used both TALENs (transcription activator‐like effector nucleases) and clustered regularly interspaced short palindromic repeats (CRISPR)/CRISPR‐associated protein 9 (Cas9) to introduce heritable base pair mutations in mature miRNA sequences. For rice, TALEN constructs were built targeting five different mature miRNA sequences and yielding heritable mutations. Among the resulting mutants, *mir390* mutant showed a severe defect in the shoot apical meristem (SAM), a shootless phenotype, which could be rescued by the wild‐type *MIR390*. Small RNA sequencing showed the two base pair deletion in *mir390* substantially interfered with miR390 biogenesis. In Arabidopsis, CRISPR/Cas9‐mediated editing of the miR160* strand confirmed that the asymmetric structure of miRNA is not a necessary determinant for secondary siRNA production. CRISPR/Cas9 with double‐guide RNAs successfully generated *mir160a* null mutants with fragment deletions, at a higher efficiency than a single‐guide RNA. The difference between the phenotypic severity of *miR160a* mutants in Col‐0 versus Ler backgrounds highlights a diverged role for miR160a in different ecotypes. Overall, we demonstrated that TALENs and CRISPR/Cas9 are both effective in modifying miRNA precursor structure, disrupting miRNA processing and generating miRNA null mutant plants.

## Introduction

MicroRNAs (miRNAs) are 20‐ to 24‐nucleotide (nt) endogenous small RNAs that function mainly to suppress target gene expression by binding to complementary mRNA sequences. The biogenesis of miRNAs was thoroughly characterized in recent years (reviewed in Achkar *et al.*, 2016; Treiber *et al.*, 2019). In animals, there are three major biogenesis pathways, while in plants there is only one characterized. In general, the processing of RNA transcript into mature miRNA can be divided into four stages: pri‐miRNA (primary miRNA), pre‐miRNA, miRNA/miRNA* duplex and mature miRNA.

The pri‐miRNA is the long RNA transcribed from a *MIRNA* (*MIR*) gene by RNA polymerase II (Pol II), yielding a long, polyadenylated, single‐stranded RNA. This RNA forms a hairpin structure and is processed by DCL1 (DICER‐LIKE 1), a member of the RNase III family. The product of this process is a much shorter RNA with a stem‐loop structure named the pre‐miRNA. The pre‐miRNA stem‐loop structure usually exposes a duplex of the mature miRNA and miRNA* on the stem, allowing DCL1 to cleave the duplex out of the stem loop (Chen, 2005). Processing from pri‐miRNA to the miRNA/miRNA* duplex is performed in the nucleus in plants, while in animals, this step is performed in the cytoplasm (Budak and Akpinar, 2015; Ha and Kim, 2014). The difference in the biogenesis between plants and animals, as well as the variation in precursor processing within plants (Bologna *et al.*, 2013), supports the need to continue studies of determinants of the biogenesis process.

When cleaving the miRNA/miRNA* duplex from the pre‐miRNA, two different patterns were observed in plants (Bologna *et al.*, 2013). One pattern starts with the first cut by recognizing a 15‐ to 17‐nt dsRNA (double‐stranded RNA) region downstream of the miRNA/miRNA* and upstream of the loop structure also described in animals. The other mode is making the first cut at the distal part below a small terminal loop, which appears unique in plants. Either case, DCL1 will make the second cut at the site 21‐nt away from the first cut (Bologna *et al.*, 2013). Previous research in animals has shown that disruption of the core component in miRNA would cause a defect in miRNA processing (Ha and Kim, 2014). Since miRNA biogenesis in plants is unique from animals, it is useful to study how this process is impacted by manipulation of the precursor sequences and structures.

ta‐siRNAs (*trans*‐acting siRNAs) are another pathway unique to plants. A ta‐siRNA precursor gene is transcribed into long, polyadenylated mRNA and subsequently processed into ‘phased’ siRNAs (characterized by precise spacing of 21 or 24 nt); the ta‐siRNAs inhibit gene expression by binding to complementary mRNA sequences. The phased processing and spacing of ta‐siRNAs derive from initiation by a miRNA ‘trigger’ and generation of a double‐stranded precursor by RDR6 (RNA‐DEPENDENT RNA POLYMERASE 6). It was reported that a 22‐nt length of the miRNA trigger is crucial to produce ta‐siRNA (Chen *et al.*, 2010; Cuperus *et al.*, 2010). However, more recently, this 22‐nt length of the miRNA as a crucial aspect for secondary siRNA production (Chen *et al.*, 2010; Cuperus *et al.*, 2010) was questioned by another hypothesis that the structure of the miRNA duplex is the primary determinant of trigger activity to yield secondary siRNAs (Manavella *et al.*, 2012).

To address the aforementioned questions about miRNAs, it is important to have reliable technical tools to manipulate mature miRNA sequences and lengths. TALEN (transcription activator‐like effector nuclease) and CRISPR/Cas (clustered regularly interspaced short palindromic repeats/CRISPR‐associated proteins)‐based genome editing technologies are promising tools to precisely alter any genomic loci of interest in eukaryotes (Cermak *et al.*, 2011; Cong *et al.*, 2013; Jiang *et al.*, 2013; Jinek *et al.*, 2012; Li *et al.*, 2011; Mali *et al.*, 2013; Shan *et al.*, 2013). However, the utilization of TALENs and CRISPR/Cas9 systems to probe the function of *MIR* genes has still been limited. For example, TALENs have been used to knock out human *MIR* genes in cultured cells and mice and demonstrated to be useful tools for miRNA research in mammalian organisms (Kim *et al.*, 2013; Takada *et al.*, 2013; Uhde‐Stone *et al.*, 2014). But its application in plant miRNAs has yet to be demonstrated. Likewise, the promising potential of using CRISPR in study of plant miRNAs remains to be fully materialized. For instance, a limited number of studies demonstrating the feasibility of mutagenizing *MIR* genes have been reported (e.g. Jacobs *et al.*, 2015; Zhao *et al.*, 2016; Zhou *et al.*, 2017). Two soya bean *MIR* genes for miR1514 and miR1509 (Jacobs *et al.*, 2015), two Arabidopsis *MIR* genes for miR169a and miR827a (Zhao *et al.*, 2016), and *MIR* genes for miR408, miR528, miR815 and miR820 in rice (Zhou *et al.*, 2017) were recently targeted for mutagenesis. In this paper, we used both TALENs and CRISPR/Cas9 to create miRNA mutants and dissect the miRNA biogenesis pathway in rice and Arabidopsis. Our results demonstrate that besides their precise target editing function, TALENs also provide great versatility in target selection. The 12‐ to 24‐bp EBE length (effector binding elements) and 16‐ to 20‐bp spacer provide abundant target choice. Such versatility allowed us to design a target site right within the region corresponding to the mature miRNA sequences, which are only 20‐ to 24‐nt in length. CRISPR/Cas9, on the other hand, has emerged as an efficient tool for genome editing of *MIR* genes. The assessment of its efficacy in the editing of *MIR* genes would be of great importance for future functional studies of individual miRNAs, rather than the entire miRNA family.

## Results

### miRNA genomic DNA sequences edited by TALENs

**Figure 1:**
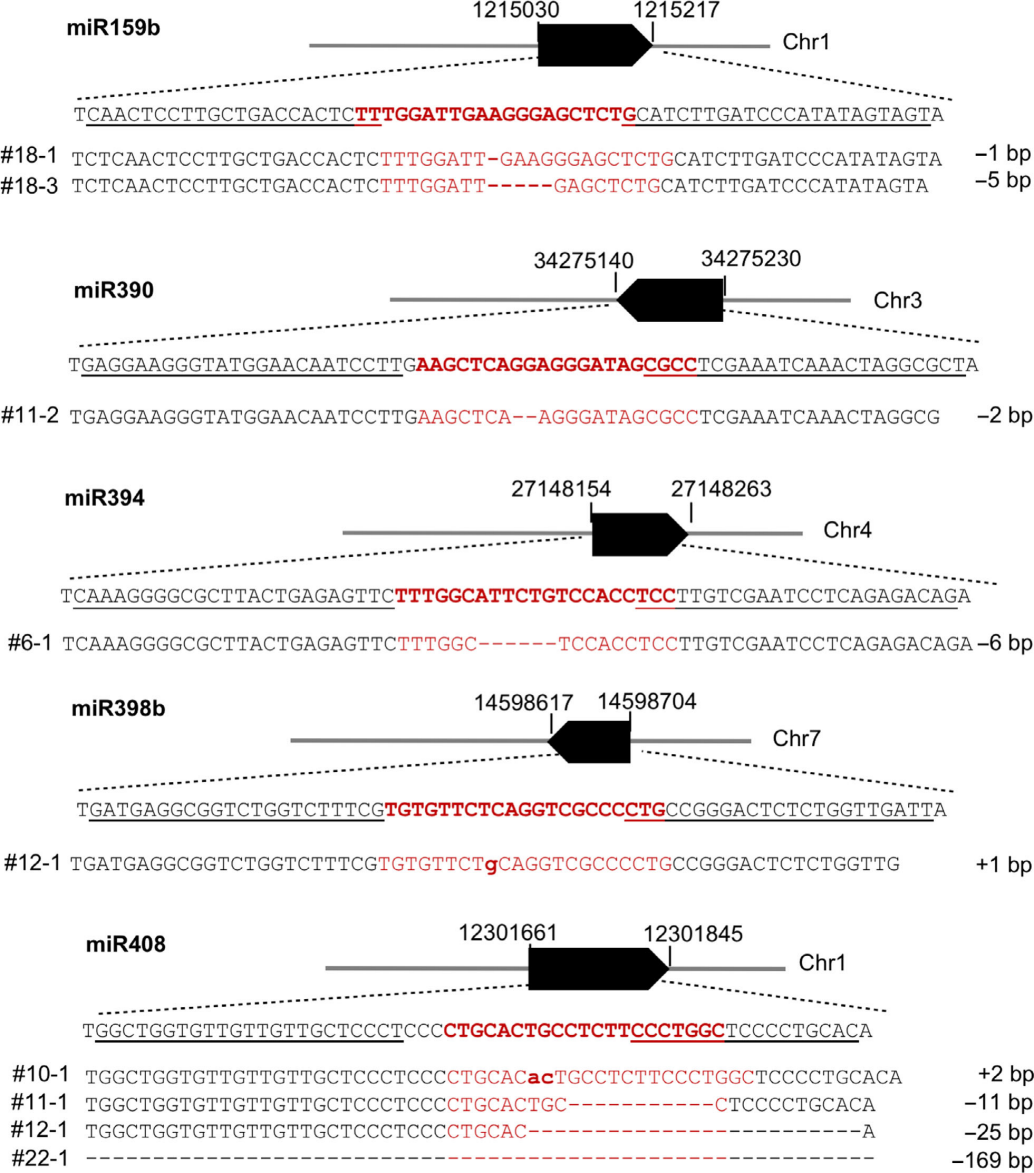
Five genomic loci targeted by TALENs and representative genotypes of mutant lines for five *MIR* genes. Underlined letters indicate sequences selected for design of paired TALENs, TALEN‐L and TALEN‐R for each *MIR* gene, while letters in red correspond to the sequences of mature miRNAs. Solid black bars denote the transcribed regions of *MIR* genes, and numbers flanking the bars are coordinates of genomic regions of *MIR* genes. All expected mutation sites were designed to be within the sequences for mature miRNAs. Individual rice lines with mutated genomic sequences aligned with their wild‐type (WT) sequences are shown under the genome loci. All mutations in T0 plants were monoallelic. Dashed lines denote nucleotides deleted and bold lower letters are for nucleotides inserted. Changes of nucleotides (insertion, + and deletion, −) are presented at the far‐right sides of sequences.

Five pairs of TALENs were designed and constructed to target five *MIR* genes (Figure 1). The transgenic plants of the first generation (T0) derived from individual constructs were screened for mutants by PCR amplification of the relevant regions and Sanger sequencing the amplicons directly. Five *MIR* genes were successfully mutated at the corresponding mature miRNA sites (red characters in Figure 1). Two lines of *mir159b* were acquired as 1‐bp and 5‐bp deletion mutations, respectively. The *mir390* mutant contained a 2‐bp deletion. *mir394* mutant contained a 6‐bp deletion and *mir398b* mutant contained a 1‐bp insertion. Four mutant lines were recovered for *MIR408* containing a 2‐bp insertion and 11‐bp, 25‐bp and 169‐bp deletions, respectively. Except for the 169‐bp deletion mutation in the *mir408* mutant, all other mutations were within the TALEN spacers. The mutated sequences that were identified are displayed in Figure 1. The inheritable mutations could be detected in the T1 and subsequent generations.

### miRNA biogenesis was disrupted by a misfolded secondary structure

As described above, miRNA biogenesis proceeds through steps from pri‐miRNA, pre‐miRNA, miRNA/miRNA* duplex and mature miRNA. The biogenesis of miRNAs relies on the stem‐loop structure of the pre‐miRNA. Since we created mutations in the mature miRNA sequences, some sequence changes could cause a slight disruption in the pre‐miRNA stem‐loop structure. In order to detect the biogenesis changes due to the altered sequences within the mature miRNA sequences, we performed small RNA sequencing in the mutants. Mutants for four out of five *MIR* genes were able to grow and produce seed. Leaves of the four mutants were harvested for small RNA library construction and sequencing.

**Figure 2:**
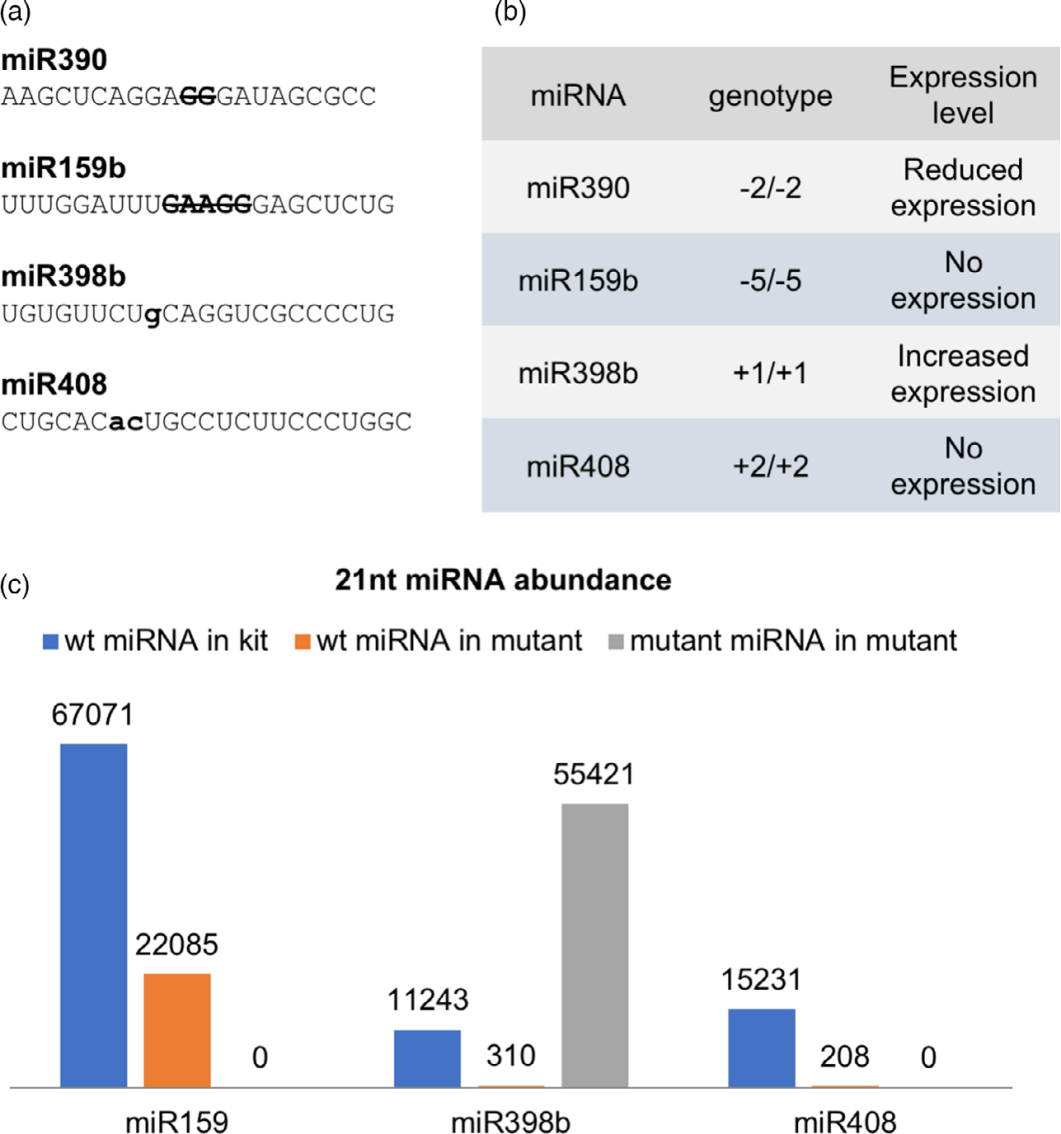
miRNA expression in *MIR* mutants. (a) Expected mature miRNA sequences in four mutant lines. Bold upper letters in strikethrough are the TALEN‐induced nucleotides, and bold lower letters represent TALEN‐introduced insertions. (b) Expression level of mature miRNA affected by TALEN‐induced mutations. The expression change in mutated alleles varies from abolished expression to increased expression. (c) 21‐nt miRNA abundance in wild type and three mutant lines. Each mutant is depicted in three columns: blue columns for wild‐type miRNA abundance in Kitaake (kit); orange columns for wild‐type miRNA abundance in mutant; grey columns for mutated miRNA abundance in mutant. The numbers above each column indicate the reads of the miRNAs.

To study whether changes in miRNA accumulation resulted from the genomic sequence change induced by TALENs, we matched small RNA reads to both wild‐type sequence and mutated sequence of miRNA (Figure 2a). The results showed that the accumulation of both miR159b and miR408 was completely abolished, while a 2‐bp deletion in miR390 caused only a reduced accumulation. However, the 1‐bp insertion in miR398b increased its accumulation level, generating an abundant 22‐nt miRNA instead of the wild‐type 21‐nt form (Figure 2 b,c). Besides the possibility that the pre‐miRNA sequence and structural change increased the biogenesis of miR398b, a feedback mechanism might also be involved. Since miR398b was mutated, the binding activity might well be decreased, which could result in an overexpression of the target gene to compensate for its reduced function.

It was reported that a 22‐nt miRNA is crucial in triggering secondary siRNA biogenesis (Chen *et al.*, 2010; Cuperus *et al.*, 2010). Yet there was also an argument that the structure of the miRNA duplex is the main determinant (Manavella *et al.*, 2012). We checked the known target sites of miR398 in rice (Os07g46990, Os04g48410 and Os03g22810), and despite the shift of the mature sequence from 21‐ to 22‐nt (which theoretically would convert it to a trigger of secondary siRNAs), there was no increase in 21‐nt siRNAs or evidence of phasing, consistent with no secondary siRNA production from these targets triggered by the 22‐nt in the *mir398b* mutant. This suggests that the 22‐nt length might not be sufficient to trigger a secondary siRNA, although it was considered a necessary condition.

### miR390 biogenesis by DCL1 was disrupted in the *mir390* mutant

**Figure 3:**
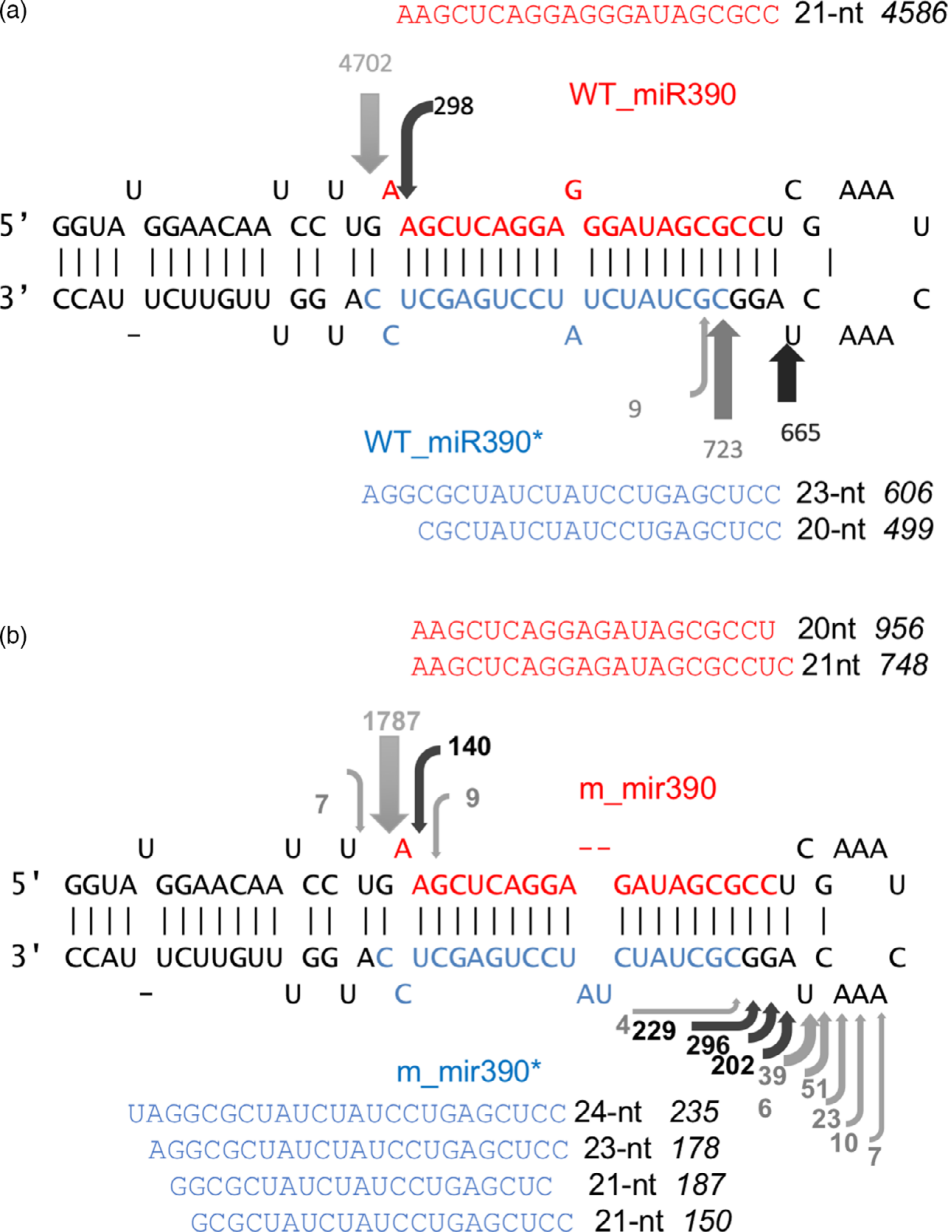
miR390 pre‐miRNA cleavage sites detected by mapped 5´ ends of small RNAs. (a) Cleavage pattern of wild‐type miR390. (b) Cleavage pattern of mutated miR390. The sequences are arranged for the predicted secondary structure of pre‐miRNA. Red letters are mature miRNA sequence, and blue letters are sequence of miRNA*. The arrows indicate the positions of the precursor cuts located below the number of reads corresponding small RNA sequences identified. The sequences besides the secondary structures show the most abundant small RNAs detected in the library. The numbers after the sequence show the length of the small RNAs, and the Italic numbers after the length indicate the abundance of small RNAs.

The sequential cleavage of pre‐miRNA into a miRNA/miRNA* duplex by DCL1 is well described (Bologna *et al.*, 2013). As mentioned above, such sequential activity can happen in both directions in plants (i.e. loop‐to‐base and base‐to‐loop), and the direction of processing varies in different miRNAs. In order to analyse the sequential cleavage of miR390, we mapped the 5´ end sequences of miRNAs in the small RNA library (Figure 3). By changing the miR390 mature miRNA sequence, cleavage activity by DCL1 was disrupted in the mutant. The loop end cleavage sites showed a clearly wider distribution, while the stem ends were preserved. Since DCL1 makes the second cuts 21‐nt away from the first cut, this result suggests the first cleavage was performed at the stem end, and the DCL1 recognition activity to generate a 21‐nt duplex length was disrupted after the first cleavage.

In order to understand how the mutation disrupted DCL1 recognition and processing, we analysed the length distribution of miR390 and miR398 in the mutants. The small RNA library data showed that the mutant miR390 occurred in length of 20‐ or 21‐nt, while the wild‐type miR390 is dominated by 21‐nt miRNAs. The miR390* in mutant showed a 21‐, 23‐ and 24‐nt predominance, while the wild type showed a 20‐ and 23‐nt dominant pattern (Figure 3). On the other hand, in the *mir398b* mutant, the 22‐nt mutant version of miR398b (UGUGUUCUgCAGGUCGCCCCUG) was dominantly produced, while the wild‐type *MIR398b* produced mostly a 21‐nt miRNA (Table S2).

DCL1 is known to typically produce 21‐nt small RNAs, while other Dicer proteins can produce small RNAs of various lengths (Pélissier *et al.*, 2011). However, the cut for the 21‐nt length of can occur on either side (or both) of the duplex, that is either the miRNA or the miRNA*. Studies have shown that the bulge structure on the miRNA/miRNA* duplex can induce variability in miRNA length while maintaining the miRNA* at 21‐nt (Cuperus *et al.*, 2010; Lee *et al.*, 2015).

Nonetheless, it is not known what determines which strand DCL1 would use to measure 21‐nt. In our study, *mir390* mutant can produce both 20‐nt and 21‐nt miRNA. Small RNA sequences of *mir390* showed the existence of a 21‐nt miRNA* that were not produced in wild‐type plants. This suggests that an unstable pre‐miRNA secondary structure in *mir390* may interfere with the recognition activity of DCL1, which thus depends on either the miRNA or miRNA* strand, depending on the pre‐miRNA secondary structure. Variation in the secondary structure could therefore change DCL1 strand selection.

### *mir390* mutants showed a severe SAM defect

**Figure 4:**
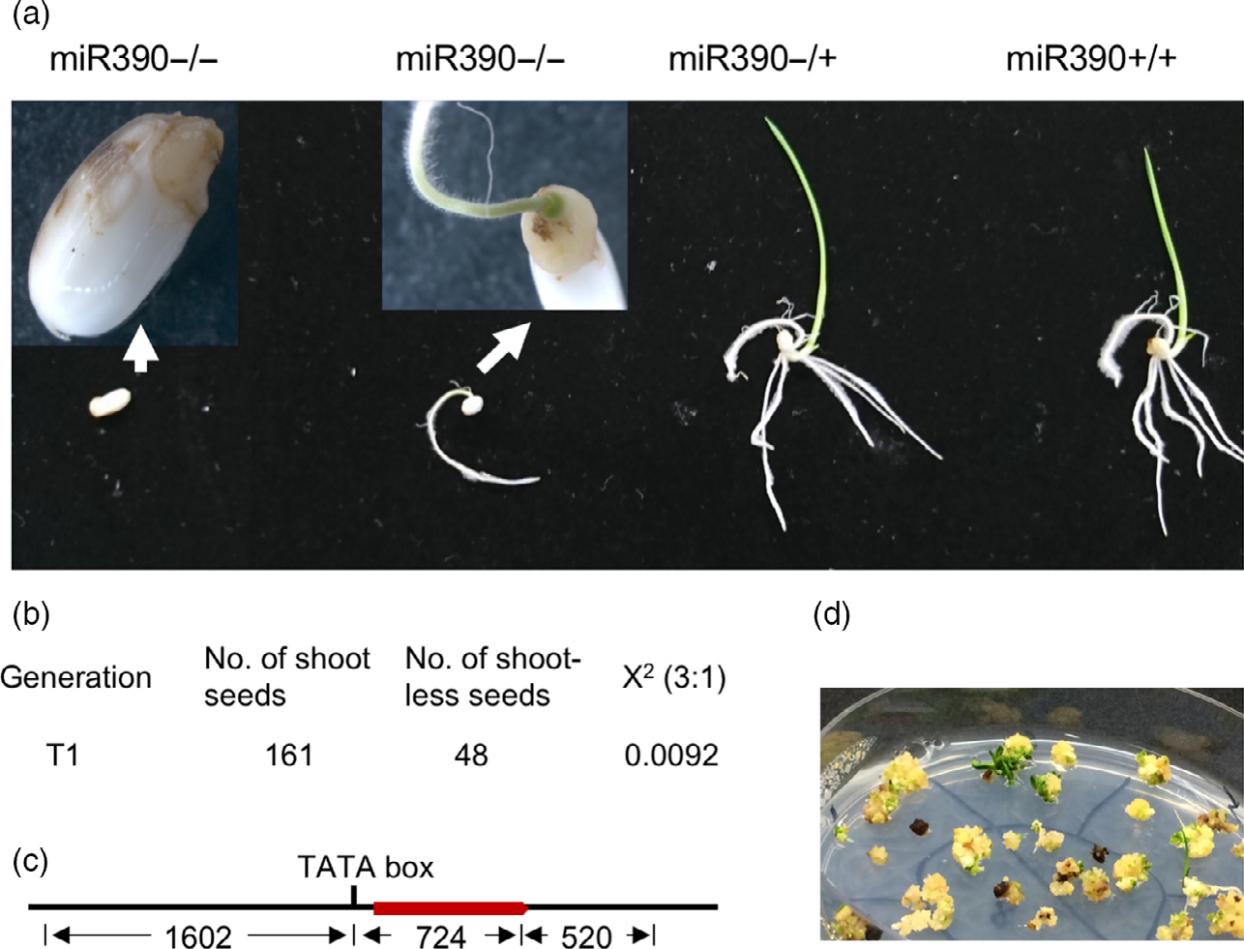
Mutation in *MIR390* causes rice shootless phenotype. (a) Shootless phenotype of *mir390*. Genotypes of germinating seeds are indicated above the picture. The mutant homozygous for miR390−/− exhibits obvious shootless phenotype while the heterozygous and wild‐type seeds show normal germination and growth. (b) Segregation ratio of miR390‐/+ progeny in germination. (c) Schematic construct of *MIR390* genomic region. Red bar represents transcribed region of *MIR390*, numbers below are lengths of corresponding region of promoter, transcribed region and terminator of *MIR390*. (d) Restoration of shoot phenotype by genomic fragment of *MIR390* from callus cells derived from the embryos of shootless mutant.

We found that heterozygous *mir390* mutant rice plants in T1 and T2 generations could not produce viable homozygous progeny plants; plus, the descendants segregated at a ratio of 1:2:1 (χ^2^ = 0.0092) as wild type: heterozygous: ungerminated seeds. Random genotyping of ungerminated seeds indicated that they were all homozygous mutants of *mir390*. The homozygous mutant seeds could either produce a root without any shoot (i.e. a SAM defect) or not germinate at all due to lack of embryonic shoot apical meristem (SAM) (χ^2^ = 0.0092, *P* = 0.92, Figure 4).

In order to confirm the effect of miR390 mutation on SAM development, we performed a rescue experiment on homozygous *mir390* (named *mir390*−/−) callus line. *mir390*−/− calli were screened and selected using the two‐step PCR method described above. The rice *MIR390* gene was PCR‐amplified as a genomic fragment and cloned into the pCambia1300 vector for complementation of *mir390*−/− mutant through rice transformation. The pCambia1300/MIR390 plasmid was then transferred into *mir390*−/− callus cells. As previously mentioned, *mir390*−/− showed a shootless phenotype. However, the transformed calli could be regenerated into plants with normal shoots, a phenotype that was rescued by the genomic fragment of *MIR390*. The sequencing results confirmed the shooting calli were indeed *mir390*−/− mutants containing a *MIR390* transgene (Figure 4).

The shootless phenotype of *mir390*−/− in our study is consistent with the phenotype observed from the *shl4* (*shootless 4*) rice mutant, which is mutant in the orthologous gene to *AGO7* in Arabidopsis. Although AGO7 and miR390 are demonstrated in Arabidopsis to be highly selective to each other, the different phenotypes of *shl4* and *ago7* in these two plant species raise questions of how they interact with miR390 differently in rice and Arabidopsis (Montgomery *et al.*, 2008; Nagasaki *et al.*, 2007). It was previously unclear whether the SAM defects in the *shl4* rice mutant were caused by *SHL4‐miR390‐TAS3* dysfunction, which would mean that the rice *TAS3* pathway is different from Arabidopsis, or perhaps there are additional unknown functions of this pathway. The result from our study showed that mutation of both *mir390* and *shl4* could cause a SAM defect in rice, demonstrating that the SAM phenotype of the *shl4* mutant is caused by *TAS3* pathway dysfunction.

### Alteration of miR160a precursor structure by CRIPSR/Cas9 in Arabidopsis

**Figure 5:**
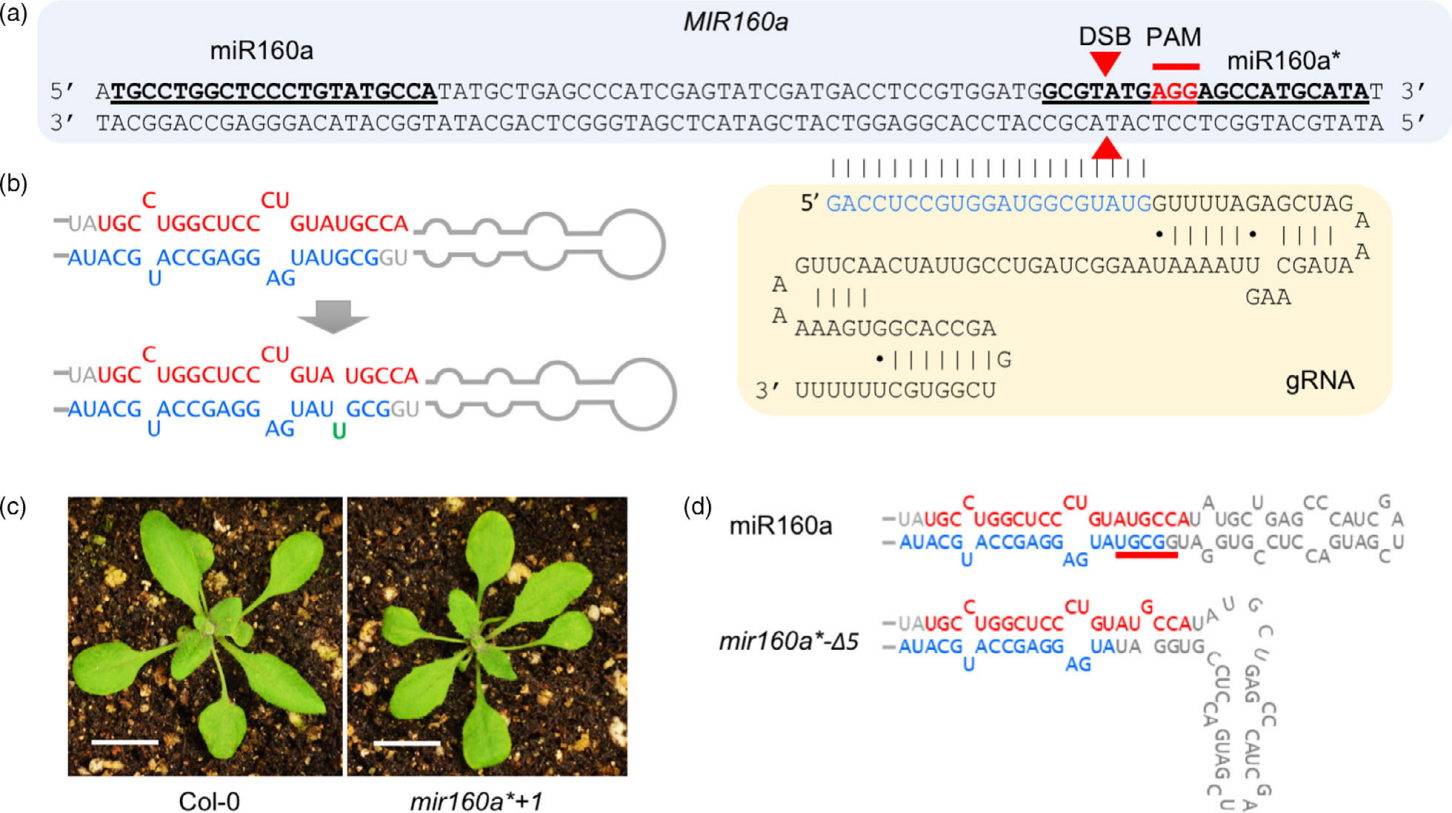
Arabidopsis *MIR160a* gene editing using CRIPSR/Cas9 with single‐guide RNA. (a) The design of CRISPR/Cas9 for the miR160a* strand. miR160 and miR160a* strands are in bold and underlined. PAM and double‐strand break (DSB) sites are labelled. The pairing sequence between the guide RNA (gRNA) and the genomic DNA is in blue. (b) The single ‘T’ insertion on the miR160a* strand makes the miRNA precursor into an asymmetric structure that produces asymmetric miRNA/miRNA* duplex. (c) The two‐week‐old wild‐type and mir160a*+1 plants. Scale bars represent 1 cm. (d) Secondary structure of miR160a precursor from wild‐type and mir160a*‐Δ5 plants. The secondary structures are redrawn based on the mfold result.

In addition to using TALENs, we attempted to edit *MIR* genes with CRISPR/Cas9, an emerging technology for genome editing; this work was done in Arabidopsis, as it is faster and a good model for other plant species. We chose *MIR160a* as the mutant phenotype has been reported (Liu *et al.*, 2010) and that it was predicted to contain multiple PAM sites on both miR160 and miR160* strands to be targeted by guide RNAs (gRNAs). We first designed a gRNA that targets the miR160* strand in order to alter the secondary structure of the miRNA precursor (Figure 5a), because (as described above) a previous study showed that a miRNA with an asymmetric duplex structure triggers secondary siRNA production from its targets with a bulge on either the miRNA or passenger (miRNA*) strand (Manavella *et al.*, 2012). A single base insertion or deletion on the miR160a* strand would result in such an asymmetric structure. Among the 17 T1 individual plants that we obtained, seven of them showed successful genome editing, confirmed by Sanger sequencing with an efficiency of ~ 41%. Among the seven mutants, six of them showed a single ‘T’ insertion at the miR160a* strand (named ‘mir160a*+1’), which would generate an asymmetric structure on the miR160a precursor and resultant miR160a/miR160a* duplex (Figure 5b). These mutants displayed consistent mild leaf serration phenotype (Figure 5c), suggesting that the single nucleotide insertion on the miR160a* strand partially inhibits DCL1 processing. To investigate whether secondary siRNAs were produced from miR160 target genes in the mir160a*+1 mutant, small RNA libraries were prepared from the leaf tissue of these lines. We found that this line bearing asymmetric miR160a precursor structure did not produce secondary siRNAs at the target genes (Figure 5d), suggesting that the secondary structure of miRNA/miRNA* duplex does not determine secondary siRNA production.

One of the T1 lines (Line 13) showed a mixed Sanger sequencing result (Figure S1a), suggesting that Line 13 contains biallelic genome edits. We continued to grow the T2 plants of Line 13 to characterize its genotype and phenotype. Sanger sequencing confirmed that the T2 population contains both single ‘T’ insertion and 5 base pair (GGCGT) deletion on the miR160a* strand (named ‘mir160a*‐Δ5’) (Figure S1a). The line mir160a*‐Δ5 is likely a null mutant, because a five base pair deletion was likely able to totally abolish the processing activity by DCL1, considering the secondary structure of miR160a precursor was substantially changed (Figure 5e). Indeed, we found that mir160a*‐Δ5 displayed a more severe phenotype of leaf serration compared with mir160a*+1 (Figure S1b). Consistent with the phenotype, qRT‐PCR confirmed that the relative expression levels of *ARF10*, *ARF16* and *ARF17*, the targets of miR160, were higher in mir160a*‐Δ5 compared with mir160a*+1 (Figure S1c). We also found that the pri‐miR160a accumulated to a much higher degree in mir160a*‐Δ5, suggesting severe inhibition of DCL1 activity. The mir160a*‐Δ5 plants also display phenotypes in flowers and siliques. The petals are inward‐curled, and that the pistils partly protrude out of the petals (Figure S1d). Moreover, we found a large proportion of unfertilized and aborted developing embryos in the silique, confirming the important role of miR160a in seed development.

### Fragment deletion on the *MIR160a* gene by CRIPSR/Cas9 with double‐guide RNAs

**Figure 6:**
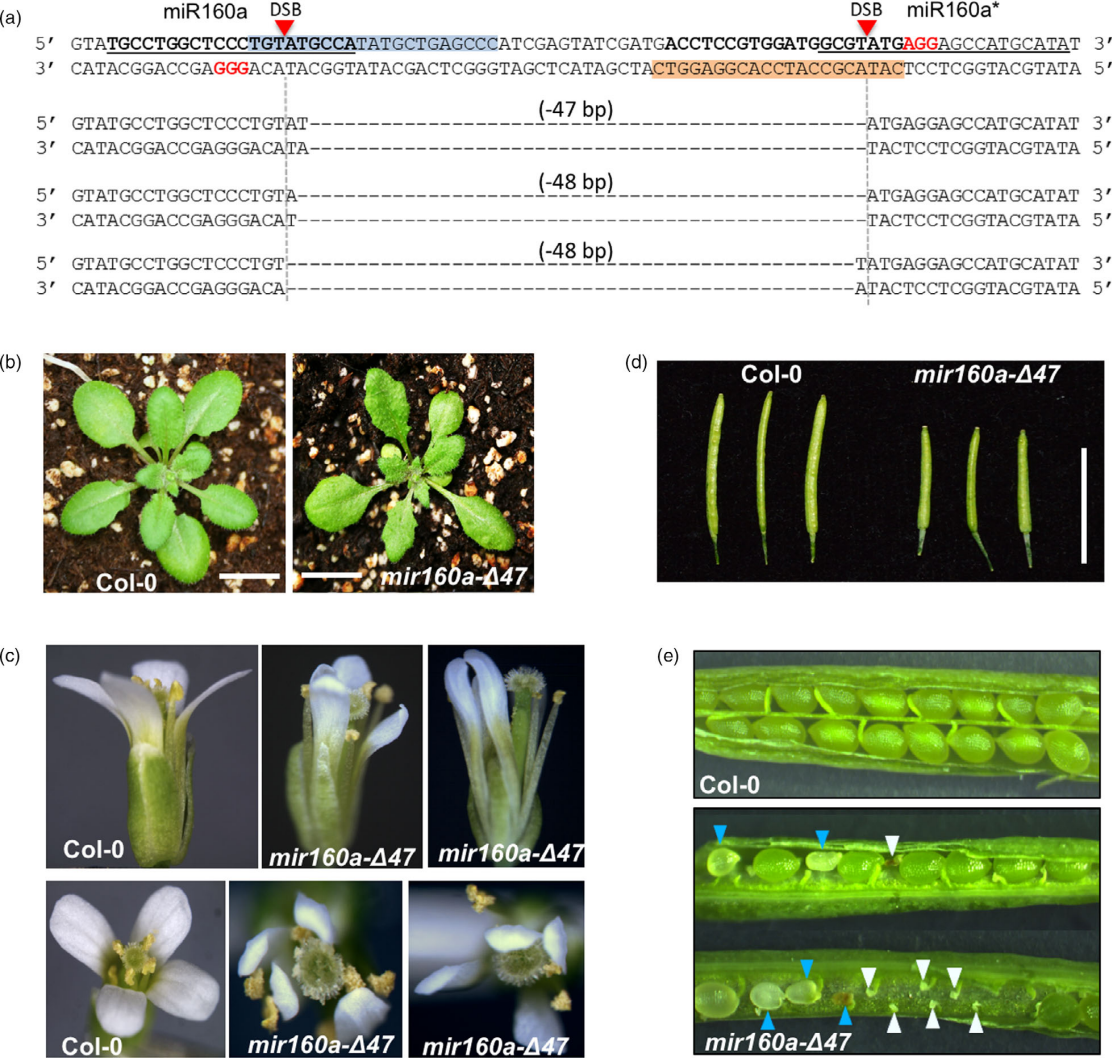
Arabidopsis *MIR160a* gene editing using CRIPSR/Cas9 with double‐guide RNA. (a) The design of CRISPR/Cas9 with double‐guide RNAs. miR160 and miR160a* strands are in bold and underlined. PAM and double‐strand break (DSB) sites are labelled. The pairing sequences between the guide RNA (gRNA) and the genomic DNA are labelled in blue and orange. The resultant 47‐ or 48‐bp fragment deletions are represented below. (b) The two‐week‐old wild‐type and *mir160a‐Δ47* plants; scale bars represent 1 cm. (c) Flower phenotypes of the *mir160a‐Δ47* mutants. (d) Siliques at the same developmental stage from wild‐type and *mir160a‐Δ47* mutants. (e) Developing seeds of the wild‐type and *mir160a‐Δ47* mutants. Blue arrows indicate delayed or aborted developing seeds; white arrows indicate unfertilized ovules.

To confirm that mir160a*‐Δ5 is a null mutant that is caused by the secondary structure change, we identified an additional PAM site on the miR160a strand, 49‐bp upstream of the aforementioned miR160a* PAM site (Figure 6a). We constructed a vector expressing double‐guide RNAs to generate miR160a mutants with fragment deletions. Among the 66 T1 plants,> 20 of them showed a discernable lower band compared with the wild‐type plants (Figure S2), indicating that genomic DNA fragment deletion occurs at ~ 30% frequency in the transformed plants.

We next obtained homozygous T2 plants with 47‐ or 48‐bp fragment deletions confirmed by Sanger sequencing (Figure 6a). These plants are null mutants, because deletion of 47‐ or 48‐bp in the miR160a precursor would eliminate the entire loop region between miR160a and miR160a* and deleted a few nucleotides from both strands. These plants display severe but developmental phenotypes consistent with mir160a*‐Δ5. For example, leaves of plants with 47‐bp deletion (named ‘*mir160a‐Δ47*’) are serrated (Figure 6b), and the petals are inward‐curled (Figure 6c). In addition, the siliques of the homozygous mutant plants are shorter than the wild‐type siliques at the same developmental stage (Figure 6d). Consistently, the homozygous mutants displayed severe defects in seed development, with a large number of unfertilized ovules and aborted or delayed developing seeds (Figure 6e). Our miR160a mutants in Col‐0 background show similar phenotypes, such as defective flower and seed development, to the *foc* mutant, which is a *Ds* transposon insertion mutant of miR160a in the Ler ecotype (Liu *et al.*, 2010). However, the mutant phenotypes in Col‐0 were milder than the published Ler phenotypes (Liu *et al.*, 2010), suggesting that the miRNA might have diversified its functions during evolution, that there might be modifying effects in the backgrounds, or that the published *Ds* transposon insertion or mutation somehow impacted the function of other genes. Further analysis of these mutants, or generating miR160a mutant in Ler using CRISPR/Cas9 would help explain this phenotypic discrepancy.

## Discussion

TALENs and CRIPSR/Cas9 are the most popular gene‐editing tools in current plant research. CRISPR/Cas9 is known for its high efficiency and easy construct assembly compared to TALENs. While TALENs require researchers to assemble the specific protein‐encoding gene by multistep reactions, the CRISPR/Cas9 system only requires a change in gRNA sequences.

On the other hand, the requirement of a PAM sequence currently limits the use of CRISPR/Cas9. In our particular application to miRNAs, for example a limitation for CRISPR/Cas9 is that most miRNAs do not contain a PAM sequence (5´‐NGG‐3´). CRISPR/Cas9 can be used to create miRNA mutants either on the miRNAs with appropriate PAM sequences, or delete the whole region by targeting both ends of the stem‐loop sequence, but internal mutations may not be possible. Deleting the whole stem loop of a miRNA should yield a null mutant, which may be useful for some analysis, such as assessing miRNA function or targets (Zhou *et al.*, 2017). But analyses of the mechanism of miRNA and secondary siRNA biogenesis are facilitated by the manipulation of internal or mature miRNA sequences instead of deleting the entire mature miRNA. The versatility of TALENs in choice of target sequences makes them a useful tool for the generation of these specific types of mutations.

We successfully created TALEN‐induced miRNA mutants with mutations specifically within mature miRNA sequences. The mutations then disrupted the miRNA biogenesis, supporting observations that mature miRNA sequences are crucial in the processing and biogenesis of miRNA precursors. Building on our success with TALENs, we also used CRISPR/Cas9 for miRNA gene editing to generate Arabidopsis mutants with either structural changes or fragment deletions. Our research demonstrated that CRISPR/Cas9 and TALENs can both be used as *MIR* gene‐editing tools.

As a result of the specific types of mutations that we produced, the mechanism of 21‐nt miRNA production by DCL1 was addressed in our study. Previous work showed that DCL1 is responsible to process most miRNAs into 21‐nt miRNA/miRNA* duplexes; this is typically done in conjunction with HYL1 (Bartel, 2004; Kurihara *et al.*, 2006). Nonetheless, there are also data demonstrating that DCL1 can produce 22‐nt miRNAs, typically when the precursor contains asymmetric bulges in the duplex such that the complementary strand still produces a 21‐nt small RNA (Cuperus *et al.*, 2010). Consistent with this, other analyses showed that DCL1 could also produce 20‐nt miRNAs when the asymmetric bulges or mismatches occur at the overhang sites (Lee *et al.*, 2015). Thus, during processing, DCL1 can select either the miRNA or miRNA* strand to measure and perform 21‐nt cleavage, with some flexibility on the length of the complementary strand; it is not clear what determines the strand selection by DCL1.

In our study, interestingly, the mutant *mir390* can produce both 20‐ and 21‐nt miRNAs, while the complementary strand showed a 21‐, 23‐ and 24‐nt dominant pattern. The wild‐type miR390 complementary strand does not produce a 21‐nt RNA. The varied miRNA lengths suggest the secondary structure of mutant pre‐miR390 loop‐stem be not as stable as wild type. Since DCL1 is typically capable of dicing miRNA* to a 21‐nt length, we infer that the 21‐nt miR390* in mutants were produced as the duplex with the 20‐nt mutant miR390, as were the 23‐ and 24‐nt miR390* variants. In other words, DCL1 could select either the miRNA or miRNA* on which to measure and perform 21‐nt dicing activity, and the selection is dependent on the secondary structure of pre‐miRNA.

In the process of editing *MIR* genes, we found that the rice *mir390*−/− mutant showed a severe shoot apical meristem defect, a shootless phenotype. Prediction algorithms for miRNA function are usually based on base matching and add a penalty for each mismatch (Fahlgren and Carrington, 2010). The data from *miR390*−/− show that the two base pair deletion could result in enormous functional change, consistent with prior observations that small but impactful changes in miRNAs or their targets can have major phenotypic consequences (Chuck *et al.*, 2007).

Previous studies in Arabidopsis showed that miR390 bond specifically to AGO7 and such binding activity demonstrated reciprocal specificity (Montgomery *et al.*, 2008). The orthologous gene of *AtAGO7* in rice, *SHL4*, was considered to have similar high selectivity to miR390. Nonetheless, the *ago7* mutant in Arabidopsis shows defects in leaf morphology instead of the severe SAM defect seen in *shl4* rice mutants (Montgomery *et al.*, 2008; Nagasaki *et al.*, 2007). Such inconsistency in phenotype may raise a question about the selectivity of miR390 and AGO7 (i.e. maybe AGO7 is required for other miRNAs, and its loss impacts multiple pathways), and their roles in regulation SAM initiation and development. In the present study, the two base pair deletion in miR390 caused severe defects in embryonic SAM development, which is consistent with *shl4* mutant phenotype. This result supports the theory that AGO7 and miR390 are highly selective to each other in both rice and Arabidopsis, that the *shl4* phenotype is solely attributed to a loss of miR390 function, and that *AGO7‐miR390‐TAS3* is crucial in rice SAM development, unlike in Arabidopsis.

Work on the rice *shl4* mutant showed that *SHL4* gene regulated expression of *HD‐ZIPIII* genes and loss of function of *SHL4* caused SAM defects (Nagasaki *et al.*, 2007)*.* The defects in the *TAS* pathway led to the up‐regulation of *ETTI/ARF* gene expression, while *miR166,* a suppressor of *HD‐ZIPIII,* increased in abundance in the mutant. It was then determined that the imbalance of *HD‐ZIPIII* and *ETTI/ARF* in the *shl4* mutant caused SAM defects in the *shl4* mutant. However, how miR390 and *SHL4* finally result in an imbalance of *HD‐ZIPIII* and *ETTI/ARF* remains unclear. This is a promising topic for future investigation.

We also generated null mutants of *miR160a* in a Col‐0 background with either altered secondary structure or deletion of the loop region of the miR160a precursor. The phenotypes of *mir160a* mutants were consistently milder than the reported *foc* mutant of miR160a in the Ler background (Liu *et al.*, 2010), suggesting that the severity of the phenotypes might be due to different genetic backgrounds. However, we could not exclude the possibility that the *foc* mutant might contain additional mutations that enhances the phenotypes. Further analysis, such as generating *mir160a* null mutants in Ler ecotype using CRISPR/Cas9 or TALEN, would possibly answer this question.

The determinant of secondary small RNA production has been controversial. It was demonstrated that the 22‐nt length of miRNAs determined secondary siRNA production (Chen *et al.*, 2010; Cuperus *et al.*, 2010). Yet, it was also argued that the structure of miRNA duplex was the determinant (Manavella *et al.*, 2012). Increasing evidence has supported the former theory; for example a miRNA that is processed as 21‐nt but uridylated to 22‐nt can trigger the secondary siRNA production (Fei *et al.*, 2018; Tu *et al.*, 2015; Zhai *et al.*, 2013); however, no direct evidence has disputed the latter theory. In our study, the 22‐nt miR398 failed to induce secondary small RNA biogenesis. On the other hand, the disruption of miR160 in Arabidopsis did not produce secondary siRNA either. Such results suggest that neither 22‐nt nor miRNA/miRNA* structure be sufficient to induce secondary small RNA production, although they might be an essential prerequisite. Further study is needed to determine the unknown factors inducing secondary siRNA productions. Again, we believe the approaches we have validated in this study will be helpful in these analyses.

With all the data above, we have demonstrated that editing mature miRNA and miRNA* sequence to disrupt pre‐miRNA stem‐loop structure would provide unique insights into mechanisms of miRNA and secondary small RNA biogenesis. To achieve this goal, both TALENs and CRISPR/Cas9 are valuable tools. Researchers need to select the proper tool accordingly. Also, the data we have provided support for previous theories, as well as some divergence conclusions. Such divergence again emphasizes the importance to subtly edit the mature miRNA sequence, compared the deleting the entire miRNA precursor. In conclusion, our research disclosed a new perspective to study biogenesis and function of miRNAs, as well as providing a set of methods to study at this direction.

## Methods

### Selection of TALEN target sites in rice genome and assembling of TALENs

TALEN targets were selected based on the rice reference genome sequence of Nipponbare (rice.plantbiology.msu.edu) using the criteria for TALEN target selection as previously described (Cermak *et al.*, 2011; Li *et al.*, 2012). Briefly with a thymine (T) preceding the EBE sequence, each EBE length ranges from 12‐ to 24‐bp. The spacer between two EBEs ranges from 16‐ to 20‐bp. In addition, the target sites were also intentionally selected so that the space sequences were located within the region corresponding to the mature miRNA sequences (Figure 1). We reasoned that TALEN‐induced changes in genomic sequence could either disrupt biogenesis of miRNA or disable it for binding to the target mRNA by changing mature miRNA sequence.

TALEN constructs were assembled as previously described (Cermak *et al.*, 2011; Li *et al.*, 2011). Briefly, the assembly kit contains 8 sets of synthesized DNA modular repeats of TAL effectors. Each set contains 4 types of modules that possess binding ability to A, T, G and C, respectively. All 8 sets have different adhesive ends matching only to their neighbouring sets in a predetermined order. Eight synthesized DNA modules corresponding to the nucleotide sequences of target DNA were then ligated together using the Golden Gate assembly method (Li and Yang, 2013), while the different adhesive ends of each module determines its position and the module member determined its binding nucleotide of target sequence. The ligated product, named an 8‐mer, contains 8 repeats with a pre‐designed order matching the 8 nucleotides of target sequence. In turn, three 8‐mers were ligated together into a modified pCambia1300 plasmid, resulting in pTALEN‐L under the *cauliflower mosaic virus* (CaMV) 35S promoter. The TALEN‐R was similarly assembled and ligated into the same pCambia1300 plasmid for expression under promoter of the maize *UBIQUITIN 1* gene. The pCambia1300 plasmid bearing both TALEN‐L and TALEN‐R was then transferred into the rice variety Kitaake (*Oryza sativa* subsp. *japonica*) to regenerate stable transgenic plants through *Agrobacterium*‐mediated transformation.

### Construction of CRISPR/Cas9 vectors

The guide RNAs for CRISPR/Cas9 were designed using the online tool CRISPR‐P (Lei *et al.*, 2014). DNA fragments containing an Arabidopsis U6 promoter and the sequence encoding guide RNAs were synthesized and digested by *PmeI*, and then inserted into the binary vector pMOA containing a plant codon‐optimized HA‐tagged nuclear‐localized (N7) Cas9 gene under the Arabidopsis *UBQ10* gene promoter, as previously described (Peterson *et al.*, 2016). The sequences of the synthesized DNA fragments are listed in Table S1.

### *Agrobacterium*‐mediated transformation

TALEN constructs were transferred to the *Agrobacterium tumefaciens* strain EHA105. Callus cells were initiated from immature embryos of Kitaake in medium containing 2‐4‐D and were cultivated with EHA105 containing the TALEN constructs accordingly to the protocol as previously described (Hiei *et al.*, 1997). Transformed calli were selected and regenerated into plantlets using hygromycin B (Sigma Aldrich, St. Louis, MI). After rooting, the plantlets were transferred to soil and grown in either a growth chamber or greenhouse. CRISPR/Cas9 constructs were transformed to *A. tumefaciens* GV3101, which was cultured overnight for floral dip transformation for Arabidopsis as previously described (Clough and Bent, 1998).

### Plant genomic DNA extraction

Plant genomic DNA extraction was performed using the CTAB method (Rogers and Bendich, 1994). Leaf tissues from plants were sampled and preserved temporally in microcentrifuge tubes on ice. Liquid nitrogen was used to freeze the tissues, which were then ground and homogenized in CTAB buffer. The homogenized tissue was in turn incubated under 55 °C for 30 min with a thorough shake every 10 min. Chloroform was added to the homogenized solution, and the mixture was then thoroughly shaken and centrifuged for 10 min under maximum speed. The supernatants after centrifugation were drawn and mixed with 0.6 volume of isopropanol, which then went through centrifugation for 10 min under maximum speed. The supernatants were discarded after centrifugation, and the precipitated pellet was washed with 80% ethanol once and air‐dried. The washed pellet containing purified plant genome DNA was dissolved in appropriate amount of HPLC‐grade water and stored under 20 °C.

### Mutation detection in plants

PCRs on targeted DNA regions were performed to identify mutations. Primers were designed to amplify 500‐ to 800‐bp DNA fragments with the pre‐selected target sites near the middle (Table S1). Sizes of PCR products were confirmed with agarose gel electrophoresis. ExoSAP‐IT PCR Product Cleanup Reagent (Thermo Fisher Scientific, Waltham, MA) was used to treat PCR products before subjected to Sanger sequencing. The Sanger sequencing trace files (chromatograms) were manually analysed for the presence or absence of and identity of mutations at the TALEN target sites.

### Two‐step PCR approach to genotyping *mir390* mutant

A two‐step PCR method was used to genotype *mir390* mutant callus line. The first step involved amplifying the *mir390* fragment containing the mutation site in the middle with primers MIR390‐F and MIR390‐R. The product from the first round PCR was diluted by 20 times and used as template for a second step PCR. The two forward primers used in the second step PCR cover the mutation site and match either wild‐type *MIR390* (MIR390‐F2) or mutant *mir390* (MIR390‐F3). Combined with a common reverse primer in the first step, both pairs of primers were used to amplify the corresponding alleles of *MIR390*. The first step PCR increases template concentration and allows more specific PCR at the second step. By the end, MIR390‐F2 gave only positive signal when there was wild‐type allele and MIR390‐F3 only functioned in the presence of a mutant allele. Genotypes (wild‐type, heterozygous mutant, homologous mutant) were determined by analysing the amplicons of the second step PCR.

### Construction of pCambia1300:MIR390

Kitaake genomic DNA was used as PCR template for high‐fidelity PCR. The genomic region of *MIR390* was PCR‐amplified with primers miR390‐F1 and miR390‐R1 (Table S1). The PCR amplicon was cloned into pENTR4 vector. In turn, the Gateway reaction was used to mobilize the *MIR390* genomic fragment into a modified pCambia1300 vector at the restriction cloning sites (EcoRI and HindIII) for transformation of *mir390*−/− derived callus cells.

### Real‐time qRT‐PCR

Total RNAs were extracted using TRIzol (Thermo Fisher Scientific). One μg of total RNA was then treated with DNase I at 37 °C for 20 min, followed by heat inactivation with EDTA. The DNA‐free RNA was then reverse‐transcribed using SuperScript III (Thermo Fisher Scientific), followed by quantitative PCR using primers as listed in Table S1. qPCR analysis was performed using the ^ΔΔ^Ct method (Livak and Schmittgen, 2001), with GAPDH as the internal control. qPCR experiments were repeated with at least 3 biological replicates to confirm consistency.

### Small RNA library construction, sequencing and analysis

Total RNAs were prepared from plant tissues using a TRIzol reagent‐based method. The plant materials were homogenized and reconstituted in TRIzol solution. The solution in turn was extracted by chloroform. Isopropanol was used to precipitate the total RNA. DEPC‐treated water was used to dissolve RNA. The total RNA underwent miRNA isolation using Qiagen miRNA purification kit according to the manufacturer’s instruction. Small RNA libraries were made using 5 µg total RNA with NEBNext Small RNA Library Prep Set as described (Mathioni *et al.*, 2017) and sequenced on the Illumina HiSeq 2500 platform. Sequencing data quality was assessed using FastQC. Reads were processed by removing the adaptor sequences using Trimmomatic (Bolger, Lohse and Usadel, 2014) and mapped to the Rice MSU genome version 7 (Kawahara *et al.*, 2013) using Bowtie (Langmead and Salzberg, 2012). Small RNA annotation was performed using miRBase v22 annotated small RNAs and Bowtie alignment software. All small RNA data were deposited in the National Center for Biotechnology Information (NCBI) Gene Expression Omnibus (GEO) under accession number GSE130510 for Arabidopsis and GSE130239 for rice.

## Conflicts of interest

The authors declare no conflict of interest.

## Author contributions

H.B., B.Y., Q.F. and B.C.M. conceived and designed the experiments. H.B., Q.F., R.L. and B.L. performed the research. R.X. and S.N.C. analysed the data. H.B. and Q.F. wrote the manuscript. B.C.M. and B.Y. revised the manuscript with input from other authors.

## Supporting information


**Figure S1 **Analysis of the *mir160a* mutants by CRIPSR/Cas9 with single‐guide RNA.
**Figure S2 **Characterization of the *mir160a* mutant with 5‐bp deletion.
**Figure S3 **Agarose gel analysis of the PCR products from CRISPR/Cas9‐transformed plants with double‐guide RNAs.
**Table S1 **Primers and synthesized DNA sequences used in this study.

**Table S2 **Small RNA library data for miRNA mutants.
