# Supplementary material for: Disruption of miRNA sequences by TALENs and CRISPR/Cas9 induces varied lengths of miRNA production
Source: Plant Biotechnol J. 2019 Dec 20;18(7):1526–36. doi: 10.1111/pbi.13315 (PMC7292542; doi:10.1111/pbi.13315)
Supplement: Supplementary file 1 — Figure S1 Analysis of the mir160a mutants by CRIPSR/Cas9 with single‐guide RNA. Figure S2 Characterization of the mir160a mutant with 5‐bp deletion. Figure S3 Agarose gel analysis of the PCR products from CRISPR/Cas9‐transformed plants with double‐guide RNAs. Table S1 Primers and synthesized DNA sequences used in this study. [file PBI-18-1526-s001.pdf]

Disruption of miRNA Sequences by TALENs and CRISPR/Cas9 Induces Varied Lengths of miRNA Production

Honghao Bi[1, ψ], Qili Fei[2,5,#,ψ], Riqing Li[1,3], Bo Liu[1,3], Rui Xia[4], Si Nian Char[1], Blake C. Meyers[3,5], Bing Yang[1,3,5,*]

[1] Department of Genetics, Development and Cell Biology, Iowa State University, Ames, IA 50011, USA

[2] Department of Plant and Soil Sciences, Delaware Biotechnology Institute, University of Delaware, Newark, DE 19711, USA

[3] Division of Plant Sciences, University of Missouri, Columbia, MO 65201, USA

[4] State Key Laboratory for Conservation and Utilization of Subtropical Agro-Bioresources, College of Horticulture, South China Agricultural University, Guangzhou, Guangdong 510642, China

[5] Donald Danforth Plant Science Center, St. Louis, MO 63132, USA

[#] Current affiliation: Guangdong Laboratory of Lingnan Modern Agriculture, Genome Analysis Laboratory of the Ministry of Agriculture, Agricultural Genomics Institute at Shenzhen, the Chinese Academy of Agricultural Sciences, Shenzhen, China.

[ψ] These authors contributed equally to this work

[*]Correspondence: yangbi@missouri.edu

Supplementary Information

(Figures S1 to S3, Table S1 and S2)

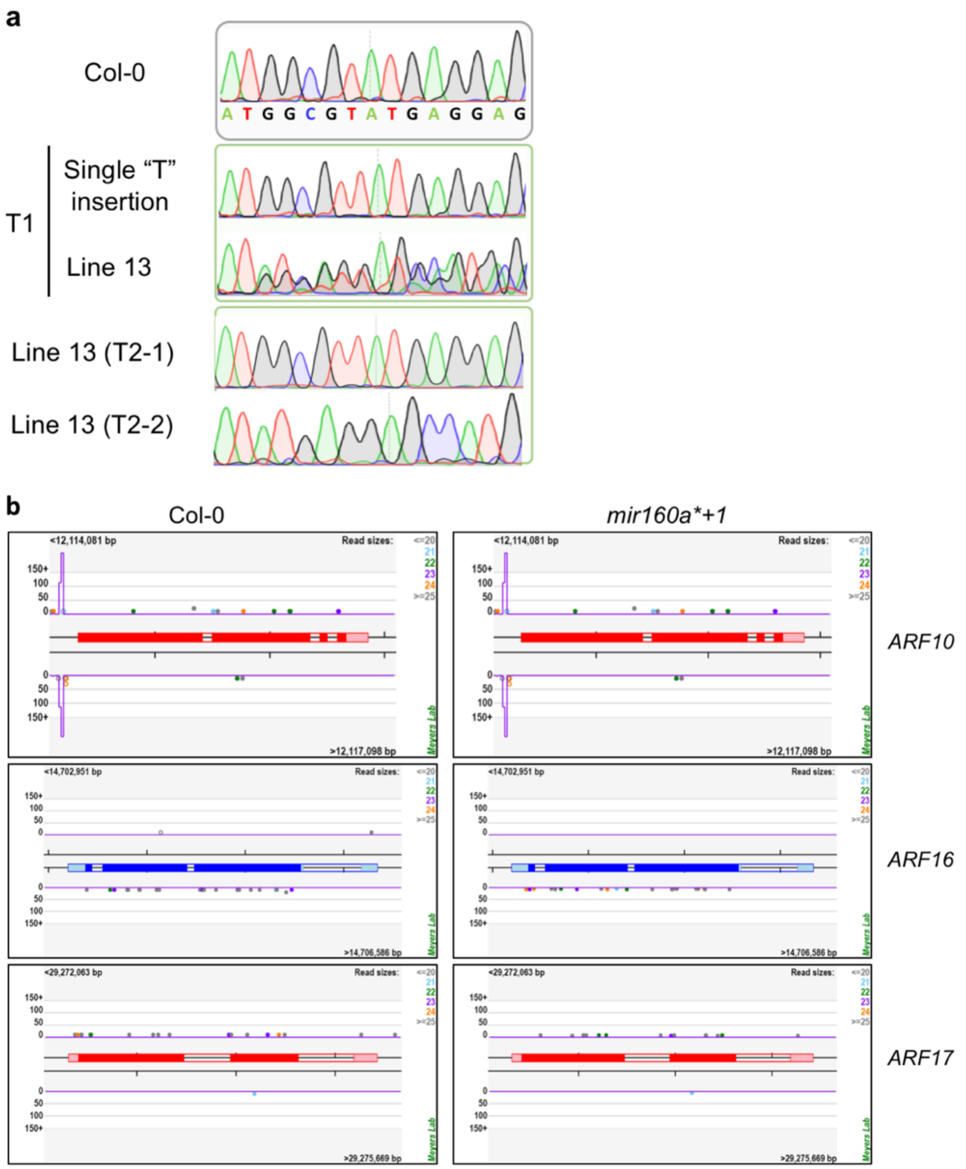

Supplementary Figure S1. Analysis of the *mir160a* mutants by CRIPSR/Cas9 with single guide RNA. **a.** Sanger sequencing results for T1 and T2 plants. Sequencing confirmed homozygous single “T” insertions and the biallelic Line 13 in T1. Sequencing results of the T2 plants of Line 13 confirmed single “T” insertion (T2-1) and 5-bp deletion (T2-2). **b.** Genome browser views of small RNAs generated from *ARF10*, *ARF16* and *ARF17* in wild type and *mir160a**+1* plants.

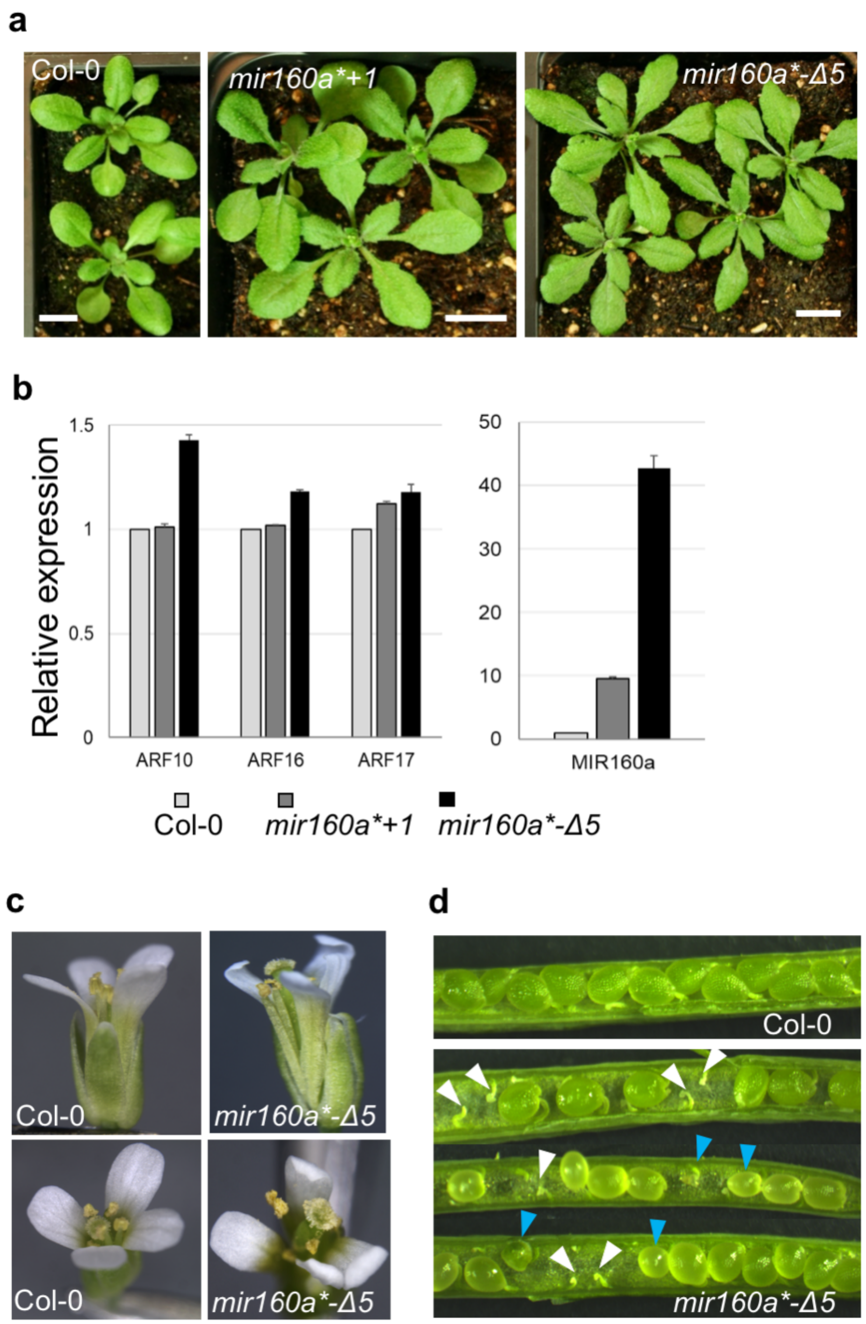

Supplementary Figure S2. Characterization of the *mir160a* mutant with 5-bp deletion.

a. The 2-3 week wild type, *mir160a*+1*, and *mir160a*-Δ5* plants; scale bars represent 1 cm. **b.** qPCR analysis of *ARF10*, *ARF16*, *ARF17*, and *PRI-MIR160A* transcript levels in wild type *mir160a*+1*, and *mir160a*-Δ5* plants. **c.** Flower phenotypes of the *mir160a*-Δ5* mutants. **d.** Developing seeds of the wild type and *mir160a*-Δ5* mutants. Blue arrows indicate delayed or aborted developing seeds; white arrows indicate unfertilized ovules.

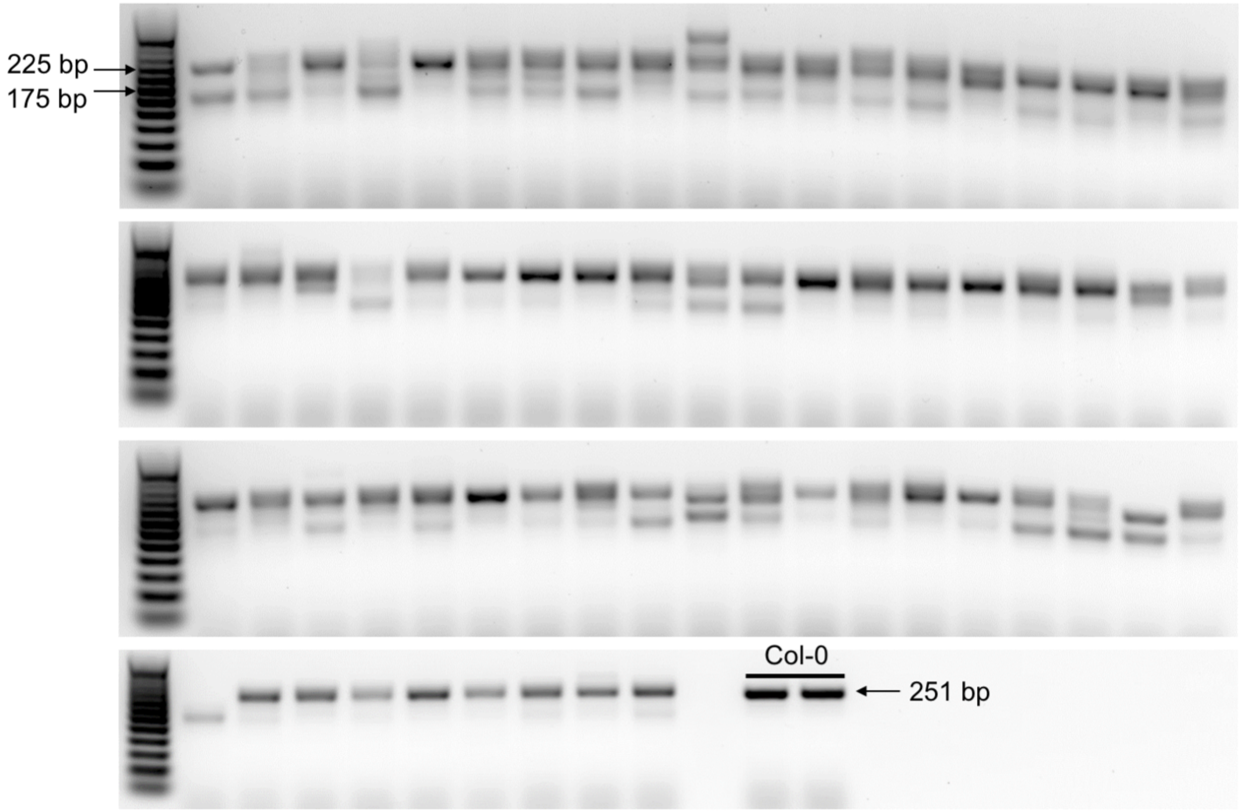

Supplementary Figure S3. Agarose gel analysis of the PCR products from CRISPR/Cas9-transformed plants with double guide RNAs. Genomic DNA of the T1 plants were extracted for PCR analysis with primers (Supplemental Table S1) to amplify the region surrounding the edit sites.

Table S1. Primers and synthesized DNA sequences used in this study

Primer name	Sequence (5' to 3')	Usage
Rice (*Oryza sativa*)		
MIR390-F	ATGGGATCCTAGAGCAAGAGGCACCACTG	PCR-amplification of *miR390* genomic region for mutant detection
MIR390-R	CATTCTAGATGCCCATCTCATGGACAGAGTAG	
miR390-F1	AATGAATTCTGACTGTACTACAAACATCCACTG	PCR-amplification of *miR390* gene for complementation
miR390-R1	TTCGGATCCAGGTACGTACTGTACAAGCTTGC	
MIR390-F2	AATCCTTGAAGCTCAGGAGGG	Forward primers for 2-step PCR-amplification of edited *miR390*
MIR390-F3	AATCCTTGAAGCTCAGGAGAT	
MIR159b-F1	ATGAATTCTCTGAAGCCTCTGATGTTTGC	Genotyping of MIR159b mutants
MIR159b-R1	CCTTCTAGATCCATCGAATATGCAGGTGCT	
MIR408-F1	ATGGAATTCAAGGCAAAGACATTGC	Genotyping of MIR408 mutants
MIR408-R1	TCATCTAGACAGCCCTTGAAGTGTCAC	
MIR394-F1	ATAGAATTCCAAGAAGCACAATCCCATC	Genotyping of MIR394 mutants
MIR394-R1	TACTCTAGAATCAACCAGGCTTGGTTAGG	
MIR398b-F1	TCATTAATCCGGTCATCAGCA	Genotyping of MIR398b mutants
MIR398b-R1	AGGTGTGCATATGTGCGTTTAC	
Arabidopsis (*Arabidopsis thaliana*)		
MIR160a-q-F	TGTCATGACGCATATACATATGTAGATG	qRT-PCR primers for *ath-MIR160a*
MIR160a-q-R	CTCATCAACACAATTCAATTGGTACC	
MIR160a-F	GATGAGGCAATGAACAGAGAC	Genotype the T1 plants edited by CRISPR/Cas9 with double guide RNA
MIR160a-R	CACCGGCGAATTAGTTTCCTTACATA	
ARF10-F	CCATTCCACGGTACTAAATTCC	qPCR primers for *ARF10*
ARF10-R	ACAAAGACGGAGATGGTGATC	
ARF16-F	CAATCTGATCAATTCGATTCCA	qPCR primers for *ARF16*
ARF16-R	GAAGATCCGAAGATGATAACCC	
ARF17-F	GTGGACTGTCTAGTGCAGCAG	qPCR primers for *ARF17*
ARF17-R	TATTGGTGAATAGCTGGGGAG	
sgRNA	GTTTAAACAAGCTTTCGTTGAACAACGGAAACTCGAC TTGCCTTCCGCACAATACATCATTTCTTCTTAGCTTTTT TTCTTCTTCTTCGTTCATACAGTTTTTTTTTGTTTATCAG CTTACATTTTCTTGAACCGTAGCTTTCGTTTTCTTCTTT TTAACTTTCCATTCGGAGTTTTTGTATCTTGTTTCATAG TTTGTCCCAGGATTAGAATGATTAGGCATCGAACCTTC AAGAATTTGATTGAATAAAACATCTTCATTCTTAAGAT ATGAAGATAATCTTCAAAAGGCCCCTGGGAATCTGAA AGAAGAGAAGCAGGCCCATTTATATGGAAAGAACA ATAGTATTTCTTATATAGGCCCATTTAAGTTGAAAACA	Synthesized DNA fragment for CRISPR/Cas9 vector construction. *Pme*I sites are highlighted in blue. The target-specific sequence of the guide RNA is highlighted in red.

	ATCTTCAAAAGTCCCACATCGCTTAGATAAGAAAACGAAGCTGAGTTTATATACAGCTAGAGTCGAAGTAGTGATTGACCUCCGUGGAUGGCGUAUGGTTTTAGAGCTAGAAATAGCAAGTTAAAATAAGGCTAGTCCGTTATCAACTTGAAAAAGTGGCACCGAGTCGGTGCTTTTTTTGTTTAAAC	
dgRNA	GTTTAAACCCCGGGCATCTTCATTCTTAAGATATGAAGATAATCTTCAAAAGGCCCCTGGGAATCTGAAAGAAGAGAAGCAGGCCCATTTATATGGGAAAGAACAATAGTATTTCTTATATAGGCCCATTTAAGTTGAAAACAATCTTCAAAAGTCCCACATCGCTTAGATAAGAAAACGAAGCTGAGTTTATATACAGCTAGAGTCGAAGTAGTGATTGGGCTCAGCATATGGCATACAGTTTTAGAGCTAGAAATAGCAAGTTAAAATAAGGCTAGTCCGTTATCAACTTGAAAAAGTGGCACCGAGTCGGTGCTTTTTTTACGTACATCTTCATTCTTAAGATATGAAGATAATCTTCAAAAGGCCCCTGGGAATCTGAAAGAAGAGAAGCAGGCCCATTTATATGGGAAAGAACAATAGTATTTCTTATATAGGCCCATTTAAGTTGAAAACAATCTTCAAAAGTCCCACATCGCTTAGATAAGAAAACGAAGCTGAGTTTATATACAGCTAGAGTCGAAGTAGTGATTGACCTCCGTGGATGGCGTATGGTTTTAGAGCTAGAAATAGCAAGTTAAAATAAGGCTAGTCCGTTATCAACTTGAAAAAGTGGCACCGAGTCGGTGCTTTTTTTGTTTAAAC	Synthesized DNA fragment for CRISPR/Cas9 vector construction with double guide RNAs. *Pme*I sites are highlighted in blue. The target-specific sequences of the guide RNA are highlighted in red.

Table S2. Small RNA library data for miRNA mutants. (please see the Excel file)

Each mutant sRNA library was screened against both wt and mutant miRNA sequence. In *miR390* mutant, heterozygous *miR390* mutant was used as control since wt rice does not contain mutant *miR390*. The result from heterozygous *miR390* mutant shows that wt allele produces significant more miRNA than mutant allele, while homozygous mutant *miR390* produces more mutant *miR390* than heterozygous mutant.
